# Ectopic Pancreatic Tissue in the Gallbladder Following Laparoscopic Cholecystectomy: A Rare Case

**DOI:** 10.7759/cureus.79445

**Published:** 2025-02-22

**Authors:** Mustafa Anil Turhan, Furkan Atakan Akin, Zumrut Merve Yasaran Benk

**Affiliations:** 1 Transplant, Memorial Ankara Hospital, Ankara, TUR; 2 General Surgery, Yildirim Beyazit University Yenimahalle Training and Research Hospital, Ankara, TUR; 3 Pathology, Sincan Training and Research Hospital, Ankara, TUR

**Keywords:** cholecystectomy, ectopic pancreas, gallbladder, heterotropy, laparoscopy, minimally invasive laparoscopy

## Abstract

Ectopic pancreatic tissue (EPT) is a rare clinical condition characterized by the presence of pancreatic tissue without any anatomical or vascular connection to the main pancreas. We aim to present a highly unusual case of EPT (fewer than 40 reported cases in the literature), located in the wall of the gallbladder. A 41-year-old female presented with episodes of right upper quadrant and epigastric pain, nausea, and vomiting. Diagnostic imaging revealed multiple gallstones, with the largest measuring 12 mm. The gallbladder wall was normal in thickness, and there were no abnormalities in the liver, bile ducts, spleen, or pancreas. Laboratory tests were normal, except for iron-deficiency anemia. Amylase and lipase levels were within normal limits. The patient underwent an uncomplicated elective laparoscopic cholecystectomy. Pathological examination of the gallbladder revealed chronic cholecystitis and EPT classified as type 3 EPT according to the Gaspar Fuentes classification. Due to its various clinical presentations and the low discriminative power of routine imaging tests, preoperative diagnosis of EPT is nearly impossible. However, considering the potential for malignant transformation and complications of EPT, physicians should be aware of this clinical entity and consider cholecystectomy when there is a high degree of suspicion. EPT in the gallbladder is an extremely rare finding. Patients are either asymptomatic or present with nonspecific symptoms, and the definitive diagnosis is almost always made through pathological examination following cholecystectomy.

## Introduction

Ectopic pancreatic tissue (EPT), also known as pancreatic heterotopia, is a rare congenital abnormality characterized by the presence of pancreatic tissue outside its normal anatomical location, without vascular or ductal connections to the pancreas. EPT is predominantly found along the gastrointestinal system (GIS), primarily in the stomach, duodenum, and colon [1]. However, it can also be found in less common sites, such as the spleen, liver, omentum, lungs, umbilicus, fallopian tube, tongue, esophagus, and gallbladder [2].

The genetic composition and physiological functions of EPT are similar to those of normal pancreatic tissue. Several theories have been proposed to explain its pathophysiology, with the "misplacement" theory being one of the most widely accepted. This theory suggests that pancreatic tissue clumps become displaced from the main pancreas into the developing gastrointestinal tract during embryogenesis [3]. Although EPT is the second most common pancreatic anomaly, its incidence in the GIS ranges from 0.55% to 13.7% in autopsy cases and 0.2% in laparotomies [1]. Incidental discoveries of EPT after stomach resections and other gastrointestinal surgeries have been reported in 0.9% and 0.2%, respectively. EPT is more frequently observed in males, with a male-to-female ratio of 3:1, and is most commonly diagnosed in their fifth and sixth decades of life [3].

The clinical significance and prevalence of gallbladder EPT remain unclear, as it is usually discovered incidentally during pathological examinations. Despite its rarity, this condition warrants consideration due to its differential diagnosis. Given that it shares histological and physiological characteristics with the pancreatic tissue, this condition should be considered because of its potential to lead to complications such as pancreatitis, obstruction, perforation, bleeding, and the risk of malignant transformation, which are not typically associated with the gallbladder. In this case, we aimed to present a highly unusual instance of EPT observed in the gallbladder wall, which is a very rare condition, with fewer than 40 cases reported in the literature. The CARE Checklist was completed by the authors for this case report.

## Case presentation

**Figure 1:**
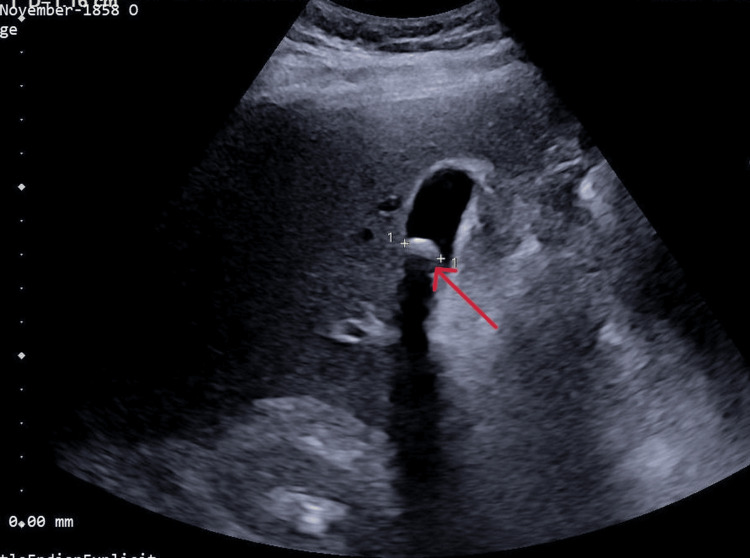
Ultrasonographic image of the patient The arrow indicates the gallbladder stone.

A 41-year-old woman presented with right upper quadrant and epigastric abdominal pain, accompanied by episodes of nausea and vomiting. There was no history of acute cholecystitis, pancreatitis, drug use, or underlying medical conditions. Diagnostic evaluations led to the diagnosis of cholelithiasis. Abdominal ultrasonography (Figure 1) revealed multiple gallstones, with the largest measuring 12 mm. The gallbladder wall thickness was normal, and no abnormalities were observed in the intrahepatic or extrahepatic bile ducts. The spleen and pancreas appeared normal in size and echotexture. Laboratory tests showed no pathological findings, except for iron deficiency anemia. The amylase and lipase levels were within normal ranges at 26 and 64 U/L, respectively. Given the symptomatic cholelithiasis, an elective laparoscopic cholecystectomy was planned and performed, with no surgical complications or difficulties encountered.

**Figure 2:**
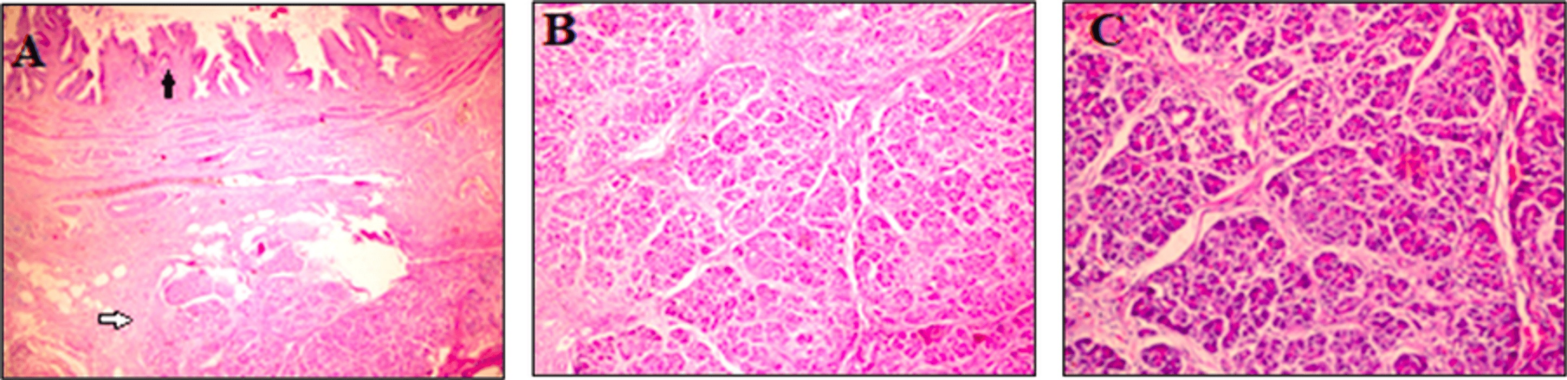
Pancreatic tissue in the histological examination of the gallbladder A: Gallbladder mucosa and underlying pancreatic lobules (H&Ex40); B: Acinar cells (H&Ex100); C: Acinar cells (H&Ex200)

The patient was discharged without any complications on the first postoperative day. Pathological examination of the resected gallbladder revealed EPT in the gallbladder wall. Pathological examination showed cholecystectomy material measuring 9.5 x 2.5 x 1.8 cm, containing multiple stones, with the largest measuring 0.5 cm in diameter. The inner surface of the gallbladder appeared macroscopically normal, without visible lesions. Microscopically, in addition to the findings of chronic cholecystitis, lobules composed of pancreatic acinar cells were present beneath the gallbladder mucosa within the submucosal and muscularis propria layers (Figure 2). Numerous samples were collected; however, no structures belonging to the ducts or endocrine pancreas were identified. Based on the Gaspar-Fuentes classification, the findings were classified as type 3 EPT [4]. The patient was placed under an early follow-up regimen with annual ultrasound examinations.

**Figure 3:**
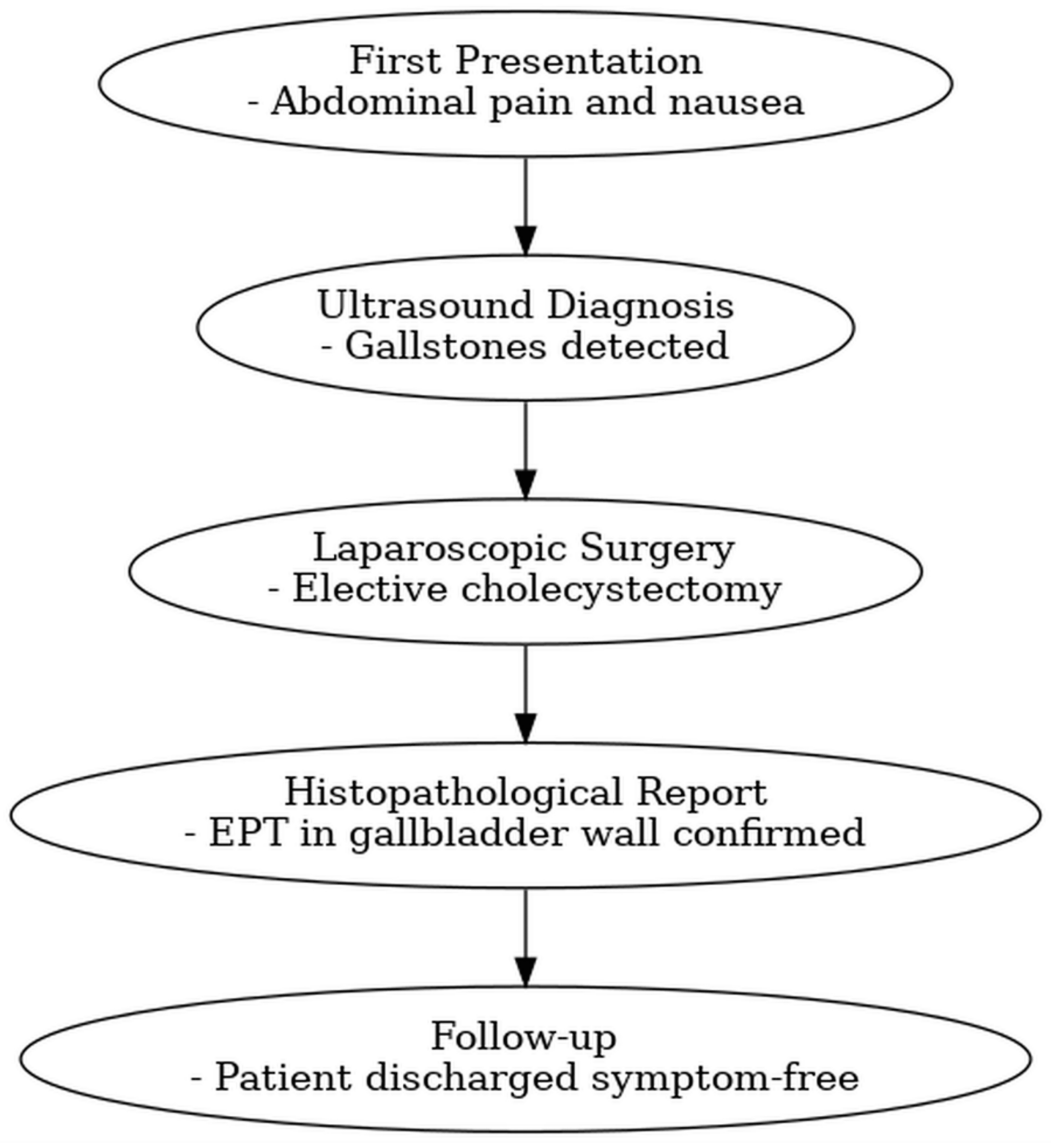
Timeline summarizing the patient’s profile History, examination, routine investigations, ultrasonography, treatment, and follow-up

The timeline of the patient's first clinical presentation to follow-up is shown in Figure 3.

## Discussion

EPT was first described by Schultz in 1727 and is commonly found in the stomach and the duodenum. In 1909, von Heinrich proposed a three-group classification system based on pathological findings, which was later modified by Fuentes et al. in 1973 [4]: type 1: acini, ducts, and islet-like pancreatic tissues; type 2: pancreatic ductular variant; type 3: exocrine pancreas with acinar tissue; and type 4: endocrine pancreas with islet cell clusters.

These classifications provide a useful framework for understanding the potential functionality and pathological behavior of EPT. In our case, histopathological examination was consistent with type 3 EPT, characterized by acinar cell clusters without significant ductal or endocrine components. Recognizing the classification of EPT can assist in evaluating possible complications such as pancreatitis, cyst formation, and tissue changes that could raise concerns for malignant transformation.

EPT is thought to result from early pancreatic separation during the embryological development of the gastrointestinal system. Another theory suggests that it arises from abnormal signaling in the foregut endoderm during embryogenesis as a result of suppression of the HES-1 transcription factor [5].

The upper gastrointestinal system is the most common site of EPT (25-38%), particularly the stomach [6]. It was previously thought that most patients had no symptoms; however, recent research has revealed that between 15% and 100% of patients exhibit symptoms, such as abdominal pain, nausea and vomiting, weight loss, and upper gastrointestinal hemorrhage. These investigations linked symptoms to factors such as age, location in the gastrointestinal system, lesion size, timing of surgery, and mucosal involvement [3].

The first case of an EPT in the gallbladder was reported by Otschkin in the early 20th century [7]. To date, fewer than 40 cases have been documented [8]. In this study, we present a case of an EPT incidentally discovered after cholecystectomy. Macroscopically, EPT in the gallbladder can resemble polypoid lesions, exophytic masses, or yellow nodules with sizes ranging from a few millimeters to 4 cm [9]. In cases of EPT in the gallbladder, it is most observed in the neck of the gallbladder [6]. Although the male-to-female ratio is 3:1 for all types of EPT, EPT in the gallbladder is predominantly found in females, likely because of the higher rate of cholecystectomy among women [10].

Gallbladder EPT can induce pathological events similar to pancreatic tissue, including the formation of cysts, pseudocysts, abscesses, and acute or chronic pancreatitis [11]. Furthermore, EPT is associated with peritonitis due to ulcerations and gallbladder perforations. It has also been speculated that enzymes released from EPT may affect and degenerate the gallbladder mucosa, potentially leading to gallbladder cancer [12]. While it is known that enzymes released from ectopic tissue can lead to neoplastic transformation, there is no evidence in the literature of a confirmed case of gallbladder carcinoma originating from a heterotopic pancreas. Nevertheless, cholecystectomy is recommended as a precautionary measure due to the potential risk of malignant transformation [13].

A study examining 54 case reports found that adenocarcinoma was the most common tumor developing in the background of EPT, predominantly located in the stomach, and most associated with Heinrich type 1 EPT [14]. Preoperative and definitive diagnoses of EPT and malignant transformation are challenging because of the location of the tissue between the submucosal and subserosal layers. A diagnosis is often made through pathological examination [15].

In addition, EPT has the potential to cause obstruction owing to mass effects. In a case reported by Kobayashi et al., an EPT located in the antrum caused duodenal obstructive stenosis, along with malignant transformation and submucosal abscess development [16]. Although similar cases involving gallbladder EPT are absent, it is possible that an EPT in Hartmann's pouch leads to symptoms resembling biliary colic by blocking the cystic duct orifice.

While radiological imaging plays a limited role in the diagnosis of EPT, recent publications have indicated that radiological techniques help in diagnosing EPT. EPTs arising within the gastrointestinal system can be identified through various examinations, such as endoscopy, endoscopic ultrasonography, computed tomography (CT), and magnetic resonance imaging (MRI). However, imaging methods may lead to confusion with other gastrointestinal conditions such as gastrointestinal stromal tumors, leiomyomas, carcinoids, neuroendocrine tumors, and intramural metastases. A definitive diagnosis was made based on histopathological examination. Although the importance of imaging techniques is recognized, especially for upper gastrointestinal EPTs, gallbladder EPTs are much rarer, and there is no gold standard for imaging methods in these cases [17]. In the absence of laboratory abnormalities, uncomplicated gallstones are not routinely investigated using techniques such as CT, MRI, or endoscopic ultrasonography. In our case, only ultrasound was used, and gallstones were detected, whereas ectopic pancreatic tissue was overlooked.

Considering this knowledge, diagnosing a gallbladder EPT can be challenging unless it presents with symptoms and complications or is incidentally discovered through imaging for other reasons. Clinicians should be careful of this condition because of its potential complications and the risk of malignant transformation that may develop in later stages. The significance of this case report lies in the presentation of another case of gallbladder EPT, an entity that could not be reliably diagnosed using preoperative imaging tests, emphasizing the importance of considering this diagnosis in clinical practice. A limitation of this study was that the patient presented with classic cholelithiasis symptoms, which did not reveal a new clinical presentation specific to EPT.

## Conclusions

EPT in the gallbladder is an extremely rare condition. Unlike ectopic pancreatic tissue found within the gastrointestinal system, the lack of distinct symptoms and inability to be diagnosed through preoperative imaging methods are situations that clinicians should be aware of, as they can potentially lead to complications and, although rare, exhibit malignant transformation owing to its characteristics similar to those of pancreatic tissue. Further data are required to determine the clinical significance and prevalence of these cases.

## References

[1] Wlaź J, Mądro A, Kaźmierak W, Celiński K, Słomka M (2014). Pancreatic and gastric heterotopy in the gastrointestinal tract. Postepy Hig Med Dosw (Online).

[2] Biswas A, Husain EA, Feakins RM, Abraham AT (2007). Heterotopic pancreas mimicking cholangiocarcinoma. Case report and literature review. JOP.

[3] Farah A, Mansour S, Khuri S (2021). Gastrointestinal tract heterotopic pancreas: asymptomatic pathology?. Gastroenterology Res.

[4] Trifan A, Târcoveanu E, Danciu M, Huţanaşu C, Cojocariu C, Stanciu C (2012). Gastric heterotopic pancreas: an unusual case and review of the literature. J Gastrointestin Liver Dis.

[5] Chatziantoniou G, Tzikos G, Ioannidis A, Loukousia A, Raptou G, Michalopoulos A, Paramythiotis D (2022). The rare entity of ectopic pancreatic tissue in the gallbladder: a case report. Ann Med Surg (Lond).

[6] Sanchiz Cárdenas EM, Soler Humanes R, Lavado Fernández AI, Díaz Nieto R, Suárez Muñoz MA (2015). Ectopic pancreas in gallbladder. Clinical significance, diagnostic and therapeutic implications. Rev Esp Enferm Dig.

[7] Neupert G, Appel P, Braun S, Tonus C (2007). [Heterotopic pancreas in the gallbladder. Diagnosis, therapy, and course of a rare developmental anomaly of the pancreas]. Chirurg.

[8] Aborajooh E, Ghayada IK, Lafi YM (2021). Heterotopic pancreas in the gallbladder: case report and literature review. Case Rep Med.

[9] Klimis T, Roukounakis N, Kafetzis I, Mouziouras V, Karantonis I, Andromanakos N (2011). Heterotopic pancreas of the gallbladder associated with chronic cholecystitis and high levels of amylasuria. JOP.

[10] Al-Shraim M, Rabie ME, Elhakeem H, Kandeel A, Shah MT, Jamil S (2010). Pancreatic heterotopia in the gallbladder associated with chronic cholecystitis: a rare combination. JOP.

[11] Elpek GO, Bozova S, Küpesiz GY, Oğüş M (2007). An unusual cause of cholecystitis: heterotopic pancreatic tissue in the gallbladder. World J Gastroenterol.

[12] Sato A, Hashimoto M, Sasaki K, Matsuda M, Watanabe G (2012). Elevation of pancreatic enzymes in gallbladder bile associated with heterotopic pancreas. A case report and review of the literature. JOP.

[13] Zaresharifi N, Khalili A, Eftekhari B, Layegh H (2023). Ectopic pancreatic tissue in a cholecystectomy specimen: a rare incidental pathologic finding. Clin Case Rep.

[14] Cazacu IM, Luzuriaga Chavez AA, Nogueras Gonzalez GM, Saftoiu A, Bhutani MS (2019). Malignant transformation of ectopic pancreas. Dig Dis Sci.

[15] Fukino N, Oida T, Mimatsu K, Kuboi Y, Kida K (2015). Adenocarcinoma arising from heterotopic pancreas at the third portion of the duodenum. World J Gastroenterol.

[16] Kobayashi S, Okayama Y, Hayashi K (2006). Heterotopic pancreas in the stomach which caused obstructive stenosis in the duodenum. Intern Med.

[17] Subramanian M, Wee E, Desai V, Peh WC (2014). Clinics in diagnostic imaging (158). Singapore Med J.

